# A Review of Federal and Statewide Guidelines and Their Effects on Orthopedics

**DOI:** 10.7759/cureus.45374

**Published:** 2023-09-16

**Authors:** Johann Braithwaite, John M Tarazi, Joshua Gruber, Jarret Boroniec, Randy Cohn, Adam Bitterman

**Affiliations:** 1 Department of Orthopedic Surgery, Zucker School of Medicine at Hofstra-Northwell Orthopedic Surgery Residency Program, Hempstead, USA; 2 Department of Orthopedic Surgery, Northwell Health-Huntington Hospital, Huntington, USA; 3 Department of Orthopedic Surgery, Dr. Kiran C. Patel College of Osteopathic Medicine, Nova Southeastern University, Fort Lauderdale, USA; 4 Department of Orthopedic Surgery, Total Orthopedics and Sports Medicine, Brooklyn, USA

**Keywords:** orthopedic surgery, state opioid regulations, prescription drug monitoring program (pdmp), monitoring opioid prescribing patterns, federal regulation, opioid utilization

## Abstract

In the past three decades, the use of opioids has risen tremendously. Pain was named the “fifth patient vital sign” in the 1990s, and from that point, opioid usage has continued to grow throughout the 2010s leading to its recognition as a crisis. The United States is responsible for 80% of the global opioid usage while only accounting for less than 5% of the global population. Previously opioids were mostly used to treat acute pain, however, opioids have been most recently used to manage chronic pain as well. The opioid crisis has presented new challenges in treating pain while preventing the abuse of these medications in a system that lacks standardization of treatment guidelines across the United States. Therefore, the authors of this review examine the current national recommendations to help manage the ongoing opioid crisis and explore how they may impact orthopedic patient care.

## Introduction and background

Declared a national public emergency in 2017, the opioid epidemic has surpassed 100,000 annual deaths and has exhibited a 600% increase since 1999 [1-3]. The rise in opioid prescriptions in the 1990s has resulted in the misuse of prescription pain relievers and many of these reported deaths [3,4]. Despite the reassurances from pharmaceutical companies to the medical community, widespread misuse continued before it became abundantly clear that these medications contained highly addictive properties [3,5]. The United States consumes nearly 80 and 99% of the global opioid and hydrocodone supplies, respectively, and the United States has additionally disproportionately contributed to the inappropriate expansion of widespread opioid use [1]. Financially, prescription opioid misuse in the United States has led to an enormous burden with healthcare and criminal justice costs amounting to $78.5 billion annually [3,6].

In 2016, the Centers for Disease Control and Prevention (CDC) released the “Guideline for Prescribing Opioids for Chronic Pain,” however, each state still maintains its autonomy regarding prescribing practices [7]. In turn, opioid prescribing laws and practices vary extensively from state to state, therefore creating a multitude of issues when combatting this crisis. Current strategies include limitations on the number of opioids prescribed, utilization of prescription drug monitoring programs (PDMPs), periodic drug screening tests, and mental health and substance abuse disorder surveys. Ultimately, the individual physician plays the most crucial role in limiting opioid misuse and abuse by abiding by state prescribing guidelines and intervening when necessary.

Orthopedic surgeons most commonly prescribe opioid medications following injury or surgery for acute pain. In 2019, a study determined that orthopedic surgeons frequently prescribed more opioid medications than were required for patients with more extensive past surgical histories [8]. Additionally, this same study suggested that up to 60% of orthopedic surgeons were not using prescription drug monitoring programs (PDMPs), and up to 79% did not provide their patients with proper instructions for the disposal of unused pills [8]. In a survey of 555 orthopedic surgeons across the United States, 42.3% admitted to having a patient whom they have prescribed opioids for, and subsequently, they developed an opioid dependency. In contrast, 35.3% admitted feeling that opioid overprescribing was not a problem in their practice [9].

Given the complexities of the current issue, it is imperative that our community examines the fundamentals of opioid medications as a form of treatment and their addictive properties that affect orthopedic patients. In doing so, the authors assess the following: (1) patient perception of pain control, (2) impact of the opioid epidemic on surgeons and patients, (3) opioid abuse prevention and prescribing strategies in the face of an opioid epidemic, 4) how varying prescribing guidelines affect orthopedic patient care, and (5) effect of online prescription drug monitoring.

## Review

Patient perception of pain control

There are several significant barriers to effective pain management. These include the following: (1) inadequate knowledge of healthcare professionals, patients, and the public, (2) poor communication between patients and providers, poor communication between providers, and lack of institutional commitment, and (3) regulatory concerns, limited access, and reimbursement for interdisciplinary care [10]. Despite the efforts by multiple national organizations, such as the Joint Commission on Accreditation of Healthcare Organizations (JCAHO), the understanding of pain management remains inconsistent. Previous studies have demonstrated overtreatment in around 30% of patients because of differences in the interpretation of pain scales between patients and providers [11]. To better understand the complex topic of patient pain control, the Hospital Consumer Assessment of Healthcare Providers and Systems (HCAHPS) survey was developed by the coordination of a group of private and government agencies and distributed to patients upon discharge from hospitals. In October 2008, the data collected from these surveys were analyzed and demonstrated a need for improvement in global patient satisfaction and pain management [12].

Gupta et al. analyzed the data from the HCAHPS survey focusing on the level of patient satisfaction with their pain control. Their analysis demonstrated that 63% of the patients surveyed were globally satisfied with pain control which highly correlated with quality of care. Additionally, the patient's relationship with healthcare staff was highly correlated with pain relief. Interestingly, government-owned hospitals accounted for the highest pain relief and privately owned hospitals accounted for the lowest pain relief. Lastly, community-level hospitals displayed the lowest satisfaction rates, while tertiary centers had the highest. Although this study evaluated data before the height of the opioid crisis, Gupta et al. provide some insight as to how subjective pain scores may correlate with patient overall satisfaction. The same researchers subsequently analyzed the HCAHPS survey report from December 2012, which showed improved patient pain control and satisfaction, however, some discrepancies were noted between the levels of hospital care, with all hospital ownership types demonstrating improved pain control [13].

Although largely subjective, accurately assessing a patient’s pain is essential. Typically, a numeric rating scale (NRS) utilizes an 11-point pain scale to measure pain severity (0=no pain and 10=worst possible pain). Oftentimes, the patient’s perceived pain score (PS) might not align with the clinical interpretation. In a 2021 prospective study, Bakshi et al. studied pain perception in post-operative patients. Patients were questioned to rate their PS on the NRS, followed by a briefing about the clinical interpretation of the scale. The patients were then rescored and differences in scores were analyzed. There was a statistically significant change in PS in 45% (162 patients), with 119 patients decreasing their post-explanation PS and 41 patients increasing their PS. However, there was no significant association with gender, time of examination, or pain management modality [14]. In conclusion, this study demonstrated the disparity in the interpretation of pain between patients and providers, therefore suggesting that it may contribute to over or under-prescribing pain medication.

The impact of the opioid epidemic on surgeons and patients

Inadequacies in treating pain in the hospital setting have been shown to lead to extended hospital stays and higher mortality [15]. Beesdo et al. demonstrated that under-treating chronic pain can lead to unnecessary hospital admissions and increased risk for patient depression, anxiety, and substance abuse [16]. Overall, inadequate pain management results in poor patient outcomes and higher hospital costs. Hence, there may be a propensity for orthopedic surgeons or providers to prescribe more medication to avoid patient dissatisfaction. Overprescribing opioids, in some cases, can lead to tolerances and worse outcomes for patients. The more significant problem emerges when tolerance evolves into dependence or addiction, with the potential for unintentional overdose deaths [17].

Orthopedic surgeons often treat patients with acute injuries and chronic conditions that require surgery. Following surgery, many of the patients receive opioid medication as part of their treatment plan to provide pain relief. In the United States, orthopedic surgeons are the fourth highest prescribers of opioids, making up an estimated 7.7% of all opioid prescriptions behind only primary care physicians (28.8%), internists (14.6%), and dentists (8.0%) [5].

A significant risk factor for post-operative opioid abuse is pre-operative opioid use. A recent study of patients who underwent total shoulder arthroplasty suggests a 3.5 times higher risk of post-operative dependence in patients who used opioids prior to their surgery with longer durations of post-operative use [18]. While not all post-operative orthopedic patients will have prolonged opioid use, greater than one to two months of use increases the risk of psychological distress and disability [19]. As such, those studies support the need for orthopedic surgeons to understand the severity of the opioid crisis and the potentially crippling effects that opioid use can have on their patients [20].

Opioid abuse prevention and prescribing strategies in the face of an opioid epidemic

The U.S. Department of Health and Human Services seeks to improve access to treatment and recovery services by promoting opioid-reversing drugs, strengthening the understanding of the crisis, promoting research on pain and addiction, and advocating for improved pain management practices. Currently, however, each state maintains its own autonomy by implementing its own prescribing practices which makes the navigation of these differences challenging [7].

The primary policy implemented by many states is to limit the number of opioid pills provided in an initial prescription (table in the Appendix) [21,22]. In 2016, Massachusetts became the first state to limit the supply of opioid prescriptions. While many states followed this precedent, it has not been uniformly adopted across the country [21]. As of 2019, 15 states have limited a seven-day supply for primary opioid prescriptions, whereas other states vary their prescribing limits by type of pain (acute versus chronic) and reasons for pain (post-surgical, neoplasm, etc.) [21]. A study of orthopedic patients in New York found that decreasing the overall number of patients prescribed opioid medications was possible by providing educational, institution-led programs [23].

The U.S. federal government has yet to require opioid analgesic prescribing limits, however, in recent years, it has released its guideline for prescribing opioids in chronic pain which includes 12 recommendations (Table 1) [7]. Even with the federal guidelines provided, 20 states have not set limits for primary opioid prescriptions (Table 2) [21]. Alabama is one state that has not set prescribing limits and consequently had the highest opioid prescribing rate per 100 people in 2018 (97.5) [24]. However, the Alabama Medicaid Agency (AMA) set in place policies for its beneficiaries similar to state legislation seen in other states meant to limit short-acting prescription opioids [25]. Conversely, Maryland has implemented a more stringent policy where physicians cannot prescribe more opioids than the lowest effective dose necessary for the patient’s condition. Although this tactic relies on the subjective decision of the provider, Maryland has accomplished an opioid prescribing rate of less than half of Alabama (45.1) [21]. While the opioid prescribing rates of these states seem to have been affected by state-set limits, the overall rate of drug overdose deaths in these states does not follow the same pattern in Alabama (30.1%) and Maryland (42.8%) [26].

**Table 1:** CDC recommendations for prescribing opioids for chronic pain outside of active cancer, palliative, and end-of-life care.

CDC recommendations for prescribing opioids for chronic pain outside of active cancer, palliative, and end-of-life care
Non-pharmacologic therapy and non-opioid pharmacologic therapy are preferred for chronic pain. Clinicians should consider opioid therapy only if expected benefits for both pain and function are anticipated to outweigh risks to the patient. If opioids are used, they should be combined with non-pharmacologic therapy and non-opioid pharmacologic therapy, as appropriate.
Before starting opioid therapy for chronic pain, clinicians should establish treatment goals with all patients, including realistic goals for pain and function, and should consider how therapy will be discontinued if benefits do not outweigh risks. Clinicians should continue opioid therapy only if there is clinically meaningful improvement in pain and function that outweighs risks to patient safety.
Before starting and periodically during opioid therapy, clinicians should discuss with patients known risks and realistic benefits of opioid therapy and patient and clinician responsibilities for managing therapy.
When starting opioid therapy for chronic pain, clinicians should prescribe immediate-release opioids instead of extended-release/long-acting (ER/LA) opioids.
When opioids are started, clinicians should prescribe the lowest effective dosage. Clinicians should use caution when prescribing opioids at any dosage, should carefully reassess evidence of individual benefits and risks when increasing dosage to 50 morphine milligram equivalents (MME)/day, and should avoid increasing dosage to 90 MME/day or carefully justify a decision to titrate dosage to 90 MME/day.
Long-term opioid use often begins with treatment of acute pain. When opioids are used for acute pain, clinicians should prescribe the lowest effective dose of immediate-release opioids and should prescribe no greater quantity than needed for the expected duration of pain severe enough to require opioids. Three days or less will often be sufficient; more than seven days will rarely be needed.
Clinicians should evaluate benefits and harms with patients within one to four weeks of starting opioid therapy for chronic pain or of dose escalation. Clinicians should evaluate benefits and harms of continued therapy with patients every three months or more frequently. If benefits do not outweigh harms of continued opioid therapy, clinicians should optimize other therapies and work with patients to taper opioids to lower dosages or to taper and discontinue opioids.
Before starting and periodically during continuation of opioid therapy, clinicians should evaluate risk factors for opioid-related harms. Clinicians should incorporate into the management plan strategies to mitigate risk, including considering offering naloxone when factors that increase risk for opioid overdose, such as history of overdose, history of substance use disorder, higher opioid dosages (50 MME/day), or concurrent benzodiazepine use, are present.
Clinicians should review the patient’s history of controlled substance prescriptions using state prescription drug monitoring program (PDMP) data to determine whether the patient is receiving opioid dosages or dangerous combinations that put him or her at high risk for overdose. Clinicians should review PDMP data when starting opioid therapy for chronic pain and periodically during opioid therapy for chronic pain, ranging from every prescription to every three months.
When prescribing opioids for chronic pain, clinicians should use urine drug testing before starting opioid therapy and consider urine drug testing at least annually to assess for prescribed medications as well as other controlled prescription drugs and illicit drugs.
Clinicians should avoid prescribing opioid pain medication and benzodiazepines concurrently whenever possible.
Clinicians should offer or arrange evidence-based treatment (usually medication-assisted treatment with buprenorphine or methadone in combination with behavioral therapies) for patients with opioid use disorder.

**Table 2:** States without limits on primary opioid prescriptions (as of 2018). *Maryland requires the “lowest effective dose.”

States without limits on primary opioid prescriptions (as of 2018)
Montana, North Dakota, Michigan, Idaho, Wyoming, South Dakota, Iowa, Illinois, Nebraska, Washington DC, Delaware, California, New Mexico, Kansas, Arkansas, Maryland*, Mississippi, Alabama, Georgia, Texas

In 2017, the U.S. Attorney General mandated that every state designate an “opioid coordinator” with the responsibilities of gathering task forces of federal, state, and local law enforcement agents to identify opioid cases for prosecution, providing legal advice and training to prosecute opioid offenses, maintaining statistics on opioid prosecutions in each district, and providing a constant evaluation of the efficacy of those strategies [2]. The results of these differences in state policies can be detected in regional differences in prescription trends in the United States. In the Northeast, for example, orthopedists prescribed at or below the median oral morphine equivalent while others in the Western and Southern states often prescribed more than the median [27].

Screening for substance abuse in the outpatient setting effectively identifies at-risk patients or those currently misusing or abusing opioids [28]. Despite the U.S. surgeon general’s recommendation for drug use screening, the literature supports that too few physicians routinely screen their patients [29]. Patients’ substance abuse can be frequently missed, however, common risk factors include a personal or family history of substance abuse, nicotine dependency, age less than 45 years, depression, and other psychiatric or personality disorders [20,30,31]. Despite knowledge of these risk factors, a recent review of 1,384 patients undergoing elective outpatient orthopedic procedures from 2018 to 2019, demonstrated that over 10% of patients were still using opioids beyond six months of their surgery [32].

Current literature has cited barriers to implementing proper screening for substance use. Given the demands of today’s medical practices, the primary challenge is allocating appropriate time for effective screening [31]. Concerted efforts to integrate substance use screening and interventions into medical practice must continue. Although this may require uncomfortable discussions for patients, identifying at-risk patients is a crucial preventative step in averting abuse or dependence. Owen et al. demonstrated that random urine drug screening on all patients with chronic opioid use might aid identification of non-adherence to the treatment plan or abuse [30].

In the United States, medical education varies widely concerning opioid prescribing and ethical training [33]. While 87% of the 102 medical schools assessed reported covering pain domains, they did so using 19 different teaching methods and eight different assessment tools [34]. Evidence-based strategies to improve opioid prescribing education include multiple medical education settings, an approach that continuously revisits and revises the topic of pain control and substance abuse, and the challenges that physicians face when prescribing these medications [33]. The Accreditation Council for Graduate Medical Education (ACGME) requires GME programs to train resident physicians and fellows to recognize and prevent addiction related to opioid use disorder [35]. Finally, there are numerous continuing medical education (CME) courses available that focus on a variety of opioid-related topics, such as handling, the stigma of addiction, overdose, prescribing and pain, and substance use in adolescence [36].

How varying prescribing guidelines affect orthopedic patient care

Orthopedic surgeons are often tasked with treating acute or chronic pain and are thus among the largest prescribers of opioid medications in the United States. While most will refer chronic pain patients to pain management specialists, the effectiveness of opioids in treating peri-operative pain has made their prescription commonplace. While opioids are effective for acute pain management, numerous studies have demonstrated that opioids can negatively impact long-term patient outcomes following procedures, such as total knee arthroplasty, reverse total shoulder arthroplasty, and spine surgery [37-39]. Patients treated with extended courses of opioids as part of post-operative care consistently report worse pain scores, increased levels of disability, and decreased overall satisfaction [37-39]. Unfortunately, orthopedic surgeons have been shown in multiple studies to prescribe more opioids than needed. Sabatino et al. determined that 61% of patients reported having unused opioids after five standard orthopedic procedures as follows: total hip and knee arthroplasty, endoscopic carpal tunnel release, arthroscopic rotator cuff repair, or lumbar decompression [40]. Although the CDC has issued guidelines for opioid treatment in chronic pain, none exist for acute pain following orthopedic surgery which may be due to the subjective nature of acute pain [7].

Orthopedic physicians can look towards other surgical specialties as a model to minimize opioid prescriptions. A 2017 study found that 60% of prescribed oxycodone-equivalent pills after urological procedures went unused [41]. It is estimated that up to 70% of pills prescribed to patients in the post-operative period for all surgical procedures go unused and most of these pills remain in the patients’ homes [2]. In another study, the same researchers recommended national procedure-specific guidelines to decrease opioid abuse in urologic patients [42]. These guidelines, which are based on a meta-analysis of opioid use in patients after urological procedures, could provide more appropriate opioid prescribing practices [42]. The same study also demonstrated that decreasing peri-operative opioid prescriptions led to decreased patient addiction habits, however, the study also showed that urologists were unlikely to change their prescribing habits [42]. It is possible that in the future, orthopedic physicians can apply the findings of this urology study or perform similar meta-analysis studies to decrease opioid addictions in their field.

An essential aspect of recovering from the opioid crisis is the education of both prescribers and patients [32-35]. More research regarding effective non-opioid management of acute traumatic pain, post-operative pain, and chronic pain management is needed for orthopedic surgery. However, multi-modal analgesia should be considered pre- and post-operatively. One of the modalities increasingly utilized in multi-modal pain management of post-operative pain is a peripheral nerve block using liposomal bupivacaine injectable suspension. Peripheral nerve blocks have been shown to decrease the need for post-operative opioids in anterior cruciate ligament (ACL) reconstruction (p=0.048) and decrease hospital stay (p<0.001), improve range of motion (p=0.008), and decrease self-reported pain scores in the first three post-operative days in total knee arthoplasty and total hip arthroplasty (p<0.001) [43,44]. While peripheral nerve blocks have been shown to help improve post-operative pain, they are not perfect and have been shown to be associated with rebound pain which has the potential to patient dissatisfaction and avoidable hospital resource utilization [45]. In addition, more research is needed to identify medical comorbidities that may propagate opioid addiction. Rather than opioids, tramadol is an alternative that has been shown to provide similar, and in many cases even better, pain relief for ACL reconstruction and arthroscopic knee debridements compared to opioids [46]. Tantillo et al. demonstrated the potential of zinc deficiency to potentiate opioid consumption, and zinc supplementation may be a simple approach to reducing opioid dependence [47]. Tantillo et al. demonstrated the potential of zinc deficiency to potentiate opioid consumption, and zinc supplementation may be a simple approach to reducing opioid dependence [47].

Effect of online prescription drug monitoring

Prescription drug monitoring programs (PDMPs) are a promising tool implemented at the state level, which allow a physician to monitor all controlled substances prescribed to a patient. Twenty percent of prescription drug users receive their prescriptions from a single prescriber. In contrast, a growing number seek their prescriptions from multiple providers, known as “doctor shopping” [48]. The tool can be essential in limiting “doctor shopping” [49]. In 1990, the first electronic PDMP was established in Oklahoma, and many states soon implemented this important tool [50]. By 2023, a PDMP will exist in every state nationally [51]. Efforts to increase PDMP utilization include expanding access to non-prescriber medical staff, integrating PDMPs into electronic medical records (EMR), and even mandated use [52].

The literature on PDMP efficacy is mixed, with some studies showing that PDMPs are ineffective at preventing or reducing opioid-related deaths [53]. However, most of these do not control for variables, such as voluntary versus mandatory usage. State regulations and workplace settings are factors that have been shown to play a role in the utilization of PDMPs [54,55]. However, favorable studies show that PDMPs have effectively reduced opioid prescribing by physicians [56].

Effective use of PDMPs can also have other positive influences on patient care. In one study, 54% of providers surveyed stated that they had made mental health or substance abuse referrals based on PDMP review, even in the setting of patients who were unlikely to ask for help when opioid abuse or dependence was demonstrated [57]. The use of PDMP data to identify a need for treatment or other healthcare needs is an essential step in the patient care plan [52]. Some states, such as Kentucky, include treatment locators in their PDMPs that show up-to-date treatment program options and availability [58]. However, until there is a federal PDMP, the benefits of the tools available on the PDMP will continue to vary by the state’s policy. National Governors Association highlights PDMP state-by-state regulatory language [52].

## Conclusions

Orthopedic surgeons are often tasked with treating patients with acute or chronic pain. Prescribing patterns by orthopedic surgeons have certainly contributed to the opioid crisis amid inconsistent state or federal regulation. The opioid crisis remains a difficult challenge to overcome; nevertheless, strides must be undertaken at federal and state levels to standardize practices involving opioid management, and prescribers must reflect on their prescribing habits and lead the efforts to overcome their contribution to the crisis.

## Appendices

**Table 3:** Opioid prescription limits compared with opioid prescribing rates by states. *Up to 14 days following surgical procedures. **Requires the "lowest effective dose." ***Limit for acute dental or ophthalmic pain. ****Up to seven days for post-operative relief. MME: morphine milligram equivalents

State	Statutory limit on opioid prescriptions	2018 opioid prescribing rate per 100
Alaska	7 days	44.9
Alabama	No limits	97.5
Arkansas	No limits	93.5
Arizona	5 days* and MME	50.7
California	No limits	35.1
Colorado	7 days	45.1
Connecticut	7 days	43.0
District of Columbia	No limits	25.0
Delaware	No limits	60.6
Florida	3-4 days	53.7
Georgia	No limits	63.2
Hawaii	7 days	33.4
Iowa	No limits	49.3
Idaho	No limits	61.9
Illinois	No limits	45.2
Indiana	7 days	65.8
Kansas	No limits	64.3
Kentucky	3-4 days	79.5
Louisiana	7 days	79.4
Massachusetts	7 days	35.3
Maryland	No limits**	45.1
Maine	7 days and MME	48.1
Michigan	No limits	62.7
Minnesota	3-4 days***	35.3
Missouri	7 days	63.4
Mississippi	No limits	76.8
Montana	No limits	54.0
North Carolina	5 days****	61.5
North Dakota	No limits	37.4
Nebraska	No limits	50.6
Nevada	14 days and MME	55.5
New Hampshire	Direction or authorization to other entities to set limits or guidelines	46.1
New Jersey	5 days	38.9
New Mexico	No limits	49.4
New York	7 days	34.0
Ohio	Direction or authorization to other entities to set limits or guidelines	53.5
Oklahoma	7 days	79.1
Oregon	Direction or authorization to other entities to set limits or guidelines	57.3
Pennsylvania	7 days	49.9
Rhode Island	MME and direction or authorization to other entities to set limits or guidelines	43.0
South Carolina	7 days	69.2
South Dakota	No limits	42.6
Tennessee	3-4 days and MME	81.8
Texas	No limits	47.2
Utah	7 days and direction or authorization to other entities to set limits or guidelines	57.1
Virginia	Direction or authorization to other entities to set limits or guidelines	44.8
Vermont	Direction or authorization to other entities to set limits or guidelines	42.4
Washington	Direction or authorization to other entities to set limits or guidelines	49.3
Wisconsin	Direction or authorization to other entities to set limits or guidelines	45.8
West Virginia	7 days	69.3
Wyoming	No limits	57.1
